# Transcriptomic and Metabolic Profiling of Kenaf Stems under Salinity Stress

**DOI:** 10.3390/plants11111448

**Published:** 2022-05-29

**Authors:** Xia An, Jie Chen, Tingting Liu, Wenlue Li, Xiahong Luo, Lina Zou

**Affiliations:** 1 Zhejiang Xiaoshan Institute of Cotton & Bast Fiber Crops, Zhejiang Institute of Landscape Plants and Flowers, Zhejiang Academy of Agricultural Sciences, Hangzhou 311251, China; liutt@zaas.ac.cn (T.L.); liwenlue@zaas.ac.cn (W.L.); luoxh@zaas.ac.cn (X.L.); zoulina@zaas.ac.cn (L.Z.); 2 College of Plant Science and Technology, Huazhong Agricultural University, Wuhan 430070, China; lqlcj@126.com

**Keywords:** kenaf, salt stress, transcriptome, metabolome, phytohormone

## Abstract

Kenaf (*Hibiscus cannabinus* L.) is an indispensable fiber crop that faces increasing salinity stress. In previous studies regarding the molecular mechanisms of how kenaf may respond to salt stress, no metabolic evidences have been reported. Meanwhile, studies regarding kenaf stems under adverse growth conditions have not been conducted. In the present study, multiple-layer evidences including physiological, transcriptomic, and metabolic data regarding how kenaf stems were affected by the salt stress are provided, wherein the stem growth, especially the lignification process, is retarded. Meanwhile, the transcriptomic data indicated genes involved in the photosynthesis are significantly repressed while the multiple flavonoid metabolism genes are enriched. As to the metabolic data, the content variation for the growth-promotion phytohormones such as IAA and the stress-responding ones including ABA are within or without expectations, implying these phytohormones played complicated roles when the kenaf stems encounter salt stress. However, the metabolite variations did not always agree with the expression levels of corresponding key pathway genes, possibly because the metabolite could be biosynthesized or catabolized in multiple pathways. Collectively, our data may enlighten, more specifically, downstream studies on kenaf responses against salinity and other adverse conditions.

## 1. Introduction

Plants as sessile organisms are encountering continuous adverse environmental conditions, of which salt stress is one of the most serious threats to plant growth and development [1]. What is even worse, this situation has been aggravated by poor irrigation practices, a rising population, and industrial pollution [2]. The exposure of plants to extensive salt concentrations can cause hazardous consequences that lead to severe losses in crop productivity and may ruin nearly half of the production of many crops [3,4]. To cope with such detrimental effects, plants have evoked certain biochemical and molecular mechanisms in response, such as the activation of cascades of molecular networks involved in stress sensing and signal transduction. During this process, specific stress-related genes and metabolites are either enhanced or suppressed [5]. For instance, the contents of some stress response hormones, such as abscisic acid (ABA), ethylene, salicylic acid (SA), and jasmonic acid (JA), changed dramatically, whilst the levels of so-called growth-promotion hormones, such as auxin, gibberellin (GA), cytokinins (CKs), brassinosteroids (BRs), and strigolactones (SLs), are also affected [6]. More importantly, these phytohormones play sophisticated and efficient roles together, rather than acting a single biological role alone [7,8,9]. Aside from these phytohormones that play vital roles in signaling and regulating the plant’s response to salt stress, a vast range of metabolites and metabolic pathways are altered when the plants confront adverse growth conditions [10,11].

Kenaf (*Hibiscus cannabinus* L.) is a fast-growing, nonwoody multipurpose annual plant species in the family Malvaceae. The kenaf plant is the third-largest fiber crop after cotton and jute. Its fiber is applicable in numerous fields, including potting and building materials, pulp and paper industry, biomass energy, composite media, potting material, building material, filtration material, board making, and animal feed [12]. However, the fiber yield and quality of kenaf suffer from severe losses under the salinity conditions, whilst the studies regarding the molecular mechanisms of kenaf encountering this particular adverse environmental condition are less conducted. At the omics scope, the proteomics studies were performed using kenaf leaves [13,14], wherein Niu et al. [13] detected 42 altered protein spots that were separated by two-dimensional gel electrophoresis and subsequently identified by matrix-assisted laser desorption ionization time of mass spectrometry. Comparatively, Kashif et al. [14] performed a combination of cytological, physiological, and proteomic analyses and accordingly identified over one hundred differentially abundant proteins. Likewise, transcriptomic approaches were also applied to develop the differentially expressed genes (DEGs) from leaves [15] and shoot tips [16] of kenaf. These big-data outputs have enhanced our understanding towards how kenaf may act, at the transcriptional and translational levels, when encountering excess salt concentrations. Moreover, the combination of multiple omics approaches may enhance our understanding towards the molecular mechanisms of how plants may respond to adverse growth conditions. For instance, a simultaneous investigation of the proteomic and mRNA-seq data of cotton under salt stress revealed the inconsistency between the transcript and proteomic levels [17], indicating an insufficient survey of the partial realm may lead to a biased conclusion. In a recent investigation into *Brassica napus* encountered salt stress [18], a combination of transcriptomics, metabolomics, and proteomics analysis was performed, which resulted in the identification of key hormones (ABA and JA) and a pivotal timepoint (24 h) that were responsible for the salt response. Meanwhile, some critical metabolites (N-acetyl-5-hydroxytryptamine, L-Cysteine, and L-(+)-Arginine) and proteins (catalase-3, cysteine desulfurase, HSP90, and P450_97 A3) were also identified. However, only single omics approaches have been conducted in each of the above-mentioned kenaf cases, and to the best of our knowledge, no metabolomics tools have been utilized to analyze the kenaf response against salt stress. By integrating the physiological, transcriptomic, and metabolomic approaches, our data suggested the kenaf stem experienced intricated reactions when encountering the salinity stress, and the involving molecular resources in the current investigation would enlighten future in-depth probing of such an issue.

## 2. Results

### 2.1. Illumina Sequencing and Assembly

The kenaf young seedlings under normal conditions (denoted as CO—control) and salinity stresses (denoted as NA—NaCl) were separately collected in duplicate, and four cDNA libraries were correspondingly constructed from these samples. Correlation analysis indicated that transcriptomic output exhibited high consistency between the control (samples CO1 and CO2) or the stress (samples NA1 and NA2) duplications (Figure S1). The overall sequencing results were shown in Table S1, within which a total of 184,883,596 clean reads (27.74 Gb) were obtained, and 175,216 unigenes were generated from these transcriptomic data. All the raw data were submitted to the NCBI database (SRR9613936 to SRR9613939).

### 2.2. Gene Annotation and Function Classification

**Figure 1:**
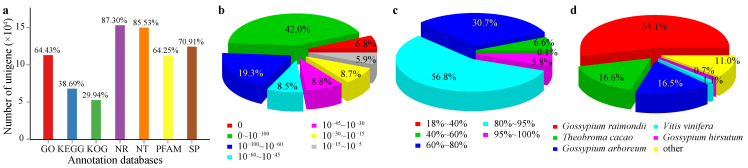
Schematic illustration of transcriptomic data annotation. The transcriptomic sequencing data were annotated against seven databases (**a**). GO, the Gene Ontology database; KEGG, the Kyoto Encyclopedia of Genes and Genomes database; KOG, the euKaryotic Ortholog Groups database; NR, the NCBI None-Redundant protein sequences database; NT, the NCBI NucleoTide sequences database; PFAM, the Protein FAMily database; SP, the Swiss-Prot database. Meanwhile, the blast results against the NR database were also presented, including distributions of e-values (**b**), sequence similarities (**c**), and best annotated species (**d**), respectively.

To gain the most possible functional information, the assembled unigenes were subjected to annotation against seven databases (Table S2). The annotation rates varied amongst the seven databases, with the highest annotation rate of 87.30% against the NCBI non-redundant (NR) database and the lowest rate of 29.94% from the euKaryotic Ortholog Groups (KOG) database. Overall, 92.52% of unigenes (162,122 of 175,216 unigenes) were annotated in at least one of the seven databases (Figure 1a). The E-values distribution against the best annotated (more unigenes mapped, Table S2 and Figure 1a) database, NR, indicated 85.4% of the blasted unigenes had E-values lower than 10^−30^ (Figure 1b), and a proportion of 93.3% of the blasted unigenes had 60% or higher sequence similarities to the blast hits (Figure 1c), which implied a high-quality annotation for our data. Meanwhile, the species classification displayed by the kenaf transcriptome genes exhibited high sequence similarity to cotton species (*Gossypium hirsutum*, 0.7%; *Gossypium raimondii*, 54.1%; *Gossypium arboreum*, 16.6%, Figure 1d). Since kenaf is a fiber crop, this attribution suggested the somehow evolutionary similarity in fiber development between kenaf and ancient cotton, especially *Gossypium raimondii*. Next, the gene functional classification was subjected to the GO, KOG, and KEGG databases. Briefly, a total of 112,897 unigenes were respectively classified into three categories (i.e., biological process, molecular function, and cellular component) against the GO database (Figure S2a), whilst fewer unigenes were annotated in the KOG (52,476 unigenes were annotated, Figure S2b) and the KEGG (67,794, Figure S2c) databases.

### 2.3. Differential Expression Analyses

**Figure 2:**
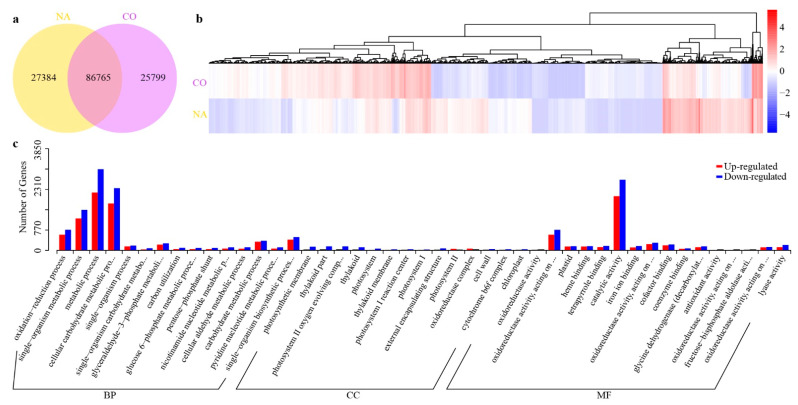
Expression analysis of unigenes. (**a**) Numbers of unigenes expressed in CO (Control) and NA (NaCl stress) conditions. (**b**) Diagram of the 10,452 differentially expressed unigenes (DEGs), the blue to red colors represents low to high relative expression levels. (**c**) GO enrichment of the up-regulated (red columns) and the down-regulated (blue columns) DEGs. BP, biological precesses; CC, cellular components; MF, molecular functions.

**Figure 3:**
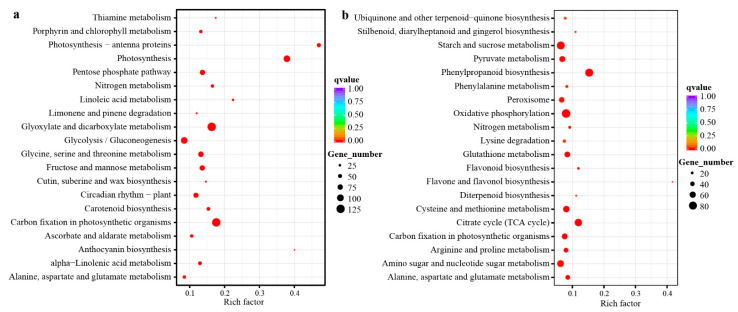
Top 20 enriched KEGG pathways among DEGs. These DEGs were classified into the down-regulated (**a**) and the up-regulated (**b**) pathways in NA samples compared with CO conditions, respectively. The circle size represents the number of enriched gene numbers, and the color is for the threshold of enrichment.

To primarily unveil the molecular mechanisms underlying the kenaf stems encountering salinity stress, the expressed unigenes between the control (denoted as CO) versus stress (NA) samples were subjected to downstream analyses. A total of 112,564 and 114,149 unigenes were respectively expressed, with FPKM (expected number of fragments per kilobase of transcript sequence per million base pairs sequenced) values of over 0.3 in the CO and NA samples, and they shared 86,765 in common (Figure 2a). Overall, 10,452 unigenes were considered to be differentially expressed genes (DEGs) between the two conditions (Figure 2b). The top GO categories enriched from the NA versus CO DEGs were “metabolic processes” in the “biological process” (BP) subfamily and “catalytic activity” in the “molecular function” (MF) subfamily (Figure 2c), indicating there may include numerous DEGs, the encoding products of which would catalyze metabolic reactions that are involved in how the kenaf seedlings respond to salt stress. These metabolic processes were likely involved in the anthocyanin biosynthesis (repressed, Figure 3a) and the flavone and flavonol biosynthesis (enhanced, Figure 3b) pathways. Meanwhile, genes included in the photosynthesis processes were significantly suppressed when the kenaf encountered salt stress (Figure 3a), and the lowest *p*-value for the enrichment of phenylpropanoid biosynthesis (Figure 3b) suggested this pathway was significantly enhanced.

### 2.4. Metabolic Profiling of Kenaf Stems

**Figure 4:**
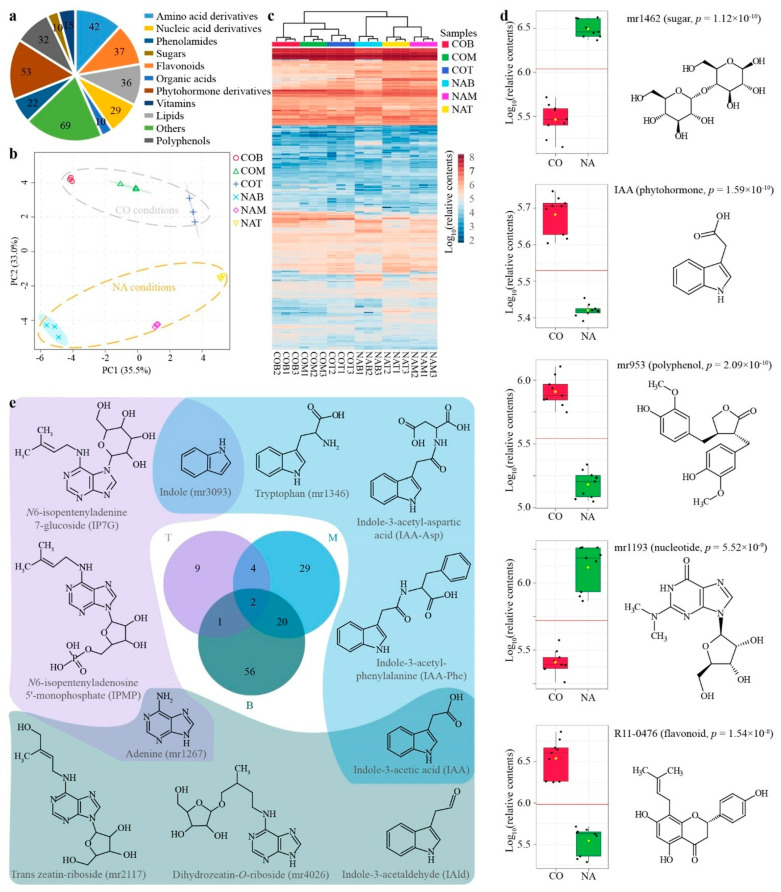
Metabolic profiling of kenaf stems. A total of 355 metabolites from various categories (**a**) have been detected. Principle component analysis of the contents variation could divide the six samples apart (**b**), which were the top (T), middle (M), and bottom (B) kenaf stems under normal (CO) or stress (NA) conditions. For instance, COT indicates the top part of stem (T) under the normal condition (CO). A more detailed contents distribution of these metabolites as a heatmap was also displayed (**c**), in which the kenaf samples could be firstly divided as control (CO) versus NaCl (NA) treatments and then as respective stem parts. The information (relative contents, *p*−value, classification, and chemical structure) of five mostly enriched metabolites in distinguishing the CO−to−NA conditions were displayed (**d**), wherein Student’s *t*-test was utilized for statistical analysis. Differentially enriched metabolites among the three stem parts were analyzed (**e**) and exemplified by the chemical structures under respectively colored backgrounds.

Since the transcriptomic output has indicated the universal transcript changes of kenaf when encountering salt stress (Figure 2 and Figure 3), we then went on to probe the alterations at the metabolic level. By applying the widely-targeted metabolomics [19] profiling protocol and utilizing a previously established metabolite library [20], a total of 355 known metabolites, containing 42 amino acids and their derivatives (AAs), 37 flavonoids (Flas), 36 lipids (Lips), 29 nucleic acids and their derivatives (NTs), 10 organic acids (Orgs), 69 Others (unclassified metabolites, Oths), 22 phenolamides (PAs), 53 phytohormones and their derivatives (PHs), 32 polyphenols (PPs), 10 sugars (Sugs), and 15 vitamins (Vits, Figure 4a, and Table S3) were detected in the kenaf stem samples that were respectively collected from the top (T), middle (M) and bottom (B) positions. The principal component analysis (PCA) of the metabolic diversity indicated major differences amongst the six kenaf stem samples (Figure 4b), in which the first principal component (PC1) explained 35.5% of the total variance, while the second (PC2) reached 33.0%. A more detailed display of the relative contents of metabolites indicated the six samples could be separated into two major groups, separating the stress conditions rather than the different stem parts (Figure 4c). This implies there may exist several key metabolites (i.e., biomarkers) that could differentiate the two growth conditions or the three stem parts. Indeed, the five most significantly enriched (ranked by *p*-values of *t*-test, Figure 4d) metabolites were mr1462 (maltose, *p* = 1.12 × 10^−10^), IAA (indole acetic acid, *p* = 1.59 × 10^−10^), mr953 (matairesinol, *p* = 2.09 × 10^−10^), mr1193 (*N2*, *N2*-dimethylguanosine, *p* = 5.52 × 10^−9^), and R11–0476 (8-prenylnaringenin, *p* = 1.54 × 10^−8^), which were respectively classified as Sugs (mr1462), PHs (IAA), PPs (mr953), NTs (mr1193), and Flas (R11–0476). The numerous classes of metabolites represented by the mostly enriched chemicals between the normal and stress conditions are indicative that, similar to the transcriptome output, widespread metabolic alterations could be observed under the salinity condition, wherein we may expect the salt stress would adjust the osmosis (sugars represented by mr1462 were elevated), repress the growth (the growth hormone IAA was decreased) and modify the lignin (the lignan mr953 and the flavonoid R11–0476 were altered, Figure 4d). Next, the differentially enriched metabolites in each of the kenaf stem samples were evaluated, which resulted in 16, 55, and 79 metabolites from the top (T), middle (M), and bottom (B) kenaf stems, respectively, at a false discovery rate (FDR) of less than 0.01 (Figure 4e and Table S4). A more careful scrutiny of these metabolites indicated the commonly enriched metabolites within two samples may derivatize different chemo-decorates that are specifically enriched in the respective samples. For instance, the metabolite mr1267 (adenine) was simultaneously enriched in T and B samples, and the zeatin metabolites (trans zeatin-riboside, mr2117, and dihydrozeatin *O*-riboside, mr4026) were found specifically enriched in the B samples, whereas the isopentenyladenine decorates (*N6*-isopentenyladenine 7-glucoside, IP7 G and *N6*-isopentenyladenosine 5′-monophosphate, IPMP) were in the T samples (Figure 4e). This may indicate the two groups of adenine-derivatized cytokinin metabolites (i.e., zeatin metabolites and isopentenyladenine decorates) acted differently in the respective kenaf stem parts when encountering salt stress. Similarly, indole-3-acetic acid (IAA) was commonly enriched in the M and B samples, while different precursors or decorates were, respectively, enriched in these two samples (Figure 4e). Considering the IAA was significantly repressed by salt stress (Figure 4d), this output implies the decreased IAA contents may be achieved respectively by amending the IAA precursors or transforming the IAA decorates into different kenaf stem parts.

### 2.5. Lignin and Phytohormone Contents, and Underlying Gene Expressions

**Figure 5:**
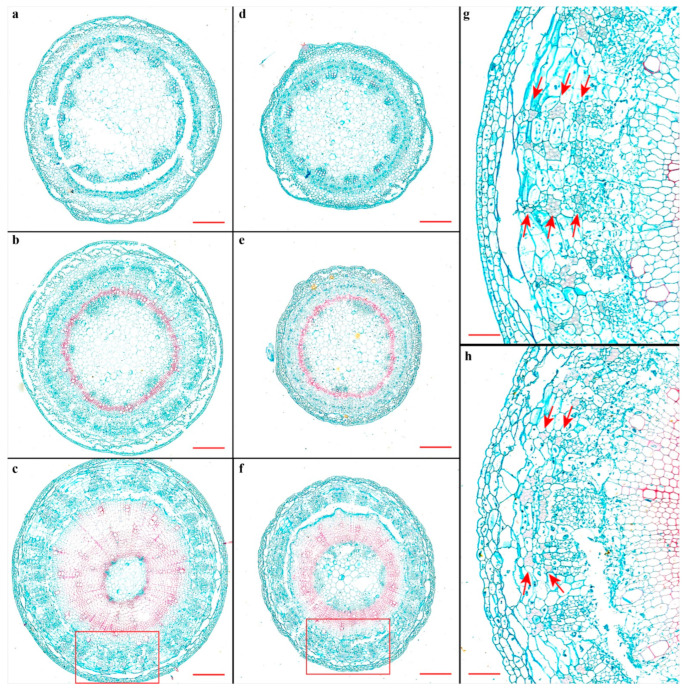
Representative slices of kenaf stems. The slices obtained from the top (**a**,**d**), middle (**b**,**e**), and bottom (**c**,**f**–**h**) parts were respectively displayed, in which the (**a**–**c**) and **g** are kenaf stem parts at normal conditions, and the rest are under salinity stress. (**g**,**h**) represent the enlarged area of the red rectangles in (**c**,**f**), respectively. The red-stained cells are lignified, and the red arrows indicated the kenaf fiber cells. Bars in (**a**) to (**f**) are 200 μm, while in (**g**,**h**) are 50 μm.

The above transcriptomic and metabolic evidences have unveiled the vast alterations of kenaf when encountering the salinity stress, wherein the phenylpropanoid-flavonoid pathway genes (Figure 3) and the flavonoid-lignan metabolites (Figure 4d) are indicative of lignin change [21,22], and the phytohormone metabolites (Figure 4e) may play sophisticated roles during this process. Indeed, the salinity stress would almost definitely lower the kenaf growth rate, which was also true for stem development and fiber formation (Figure 5). Specifically, the kenaf seedlings had decreased stem diameters (Figure 5d–f) as well as a reduced lignification process (Figure 5f) under salt stress compared with normal conditions (Figure 5a–c). Three layers of bast fiber were formed in the bottom part of CO stems (pointed by the red arrows in Figure 5g), whilst only two were observed (indicated by the red arrows in Figure 5h) under the adverse conditions. This result intuitively presented that the salinity stress had retarded the stem lignification and fiber formation.

**Figure 6:**
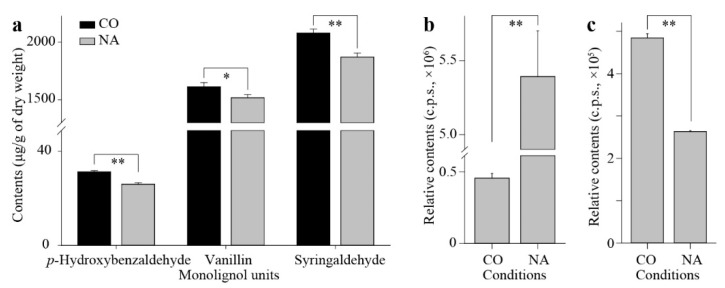
The kenaf stem growth was significantly retarded by the salt stress. Relative contents of three monolignol units (**a**), the stress hormone ABA (**b**) and the growth hormone IAA (**c**) in the whole stem at normal (CO) or adverse (NA) conditions were presented, respectively. The * or ** indicate the significant difference at threshold of 0.05 or 0.01 under Student’s *t*-test, respectively.

**Figure 7:**
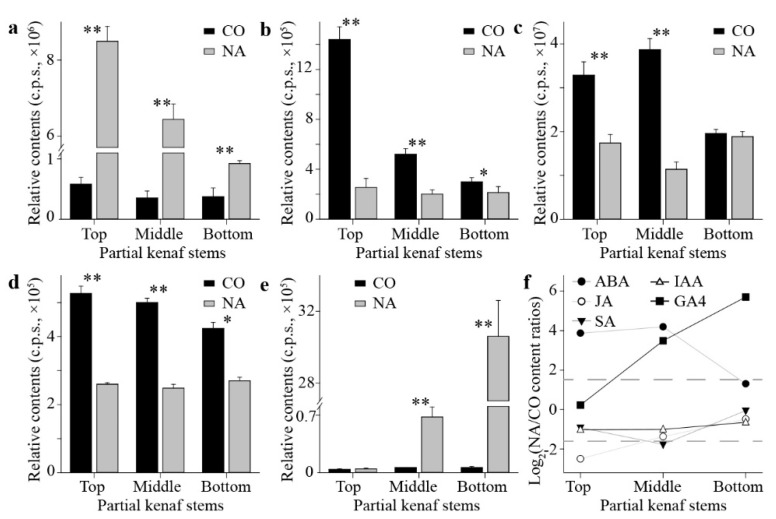
Relative contents of various phytohormones in the three kenaf stem parts at control (CO) and salt stress (NA) conditions. The relative contents of ABA (**a**), JA (**b**), SA (**c**), IAA (**d**), and GA4 (**e**) were respectively displayed. Meanwhile, the NA-to-CO contents ratio of these phytohormones was also presented (**f**). The * or ** indicate the significant difference at threshold of 0.05 or 0.01 under Student’s *t*-test, respectively.

To further quantify how the kenaf stems were affected by salt stress, the overall contents of three monolignols in the kenaf stems were measured, which showed the lignin in kenaf stems was mainly formed by G-subunits and S-subunits, and all three monolignols were universally decreased by salt stress (Figure 6a). To primarily depict how did the plant hormones react during the stress, the relative contents of phytohormones from the previously conducted metabolic profiling (Figure 4) were more specifically presented here. Correspondingly, the contents of the “stress hormone” ABA were significantly elevated (Figure 6b), whilst the “growth hormone” IAA was lowered by the salt stress (Figure 6c). The lignified part, as shown by the slicing results (red-stained area in Figure 5), was mainly formed and differentially affected at the bottom part of the kenaf stem, leading to the assumption that the underlying phytohormones acted differently amongst different parts (i.e., top, middle, and bottom parts) of the kenaf stems. The in-depth exploration of the relative contents of stress hormones (ABA, JA, and SA, Figure 7a–c) and growth hormones (IAA and GA4, Figure 7d,e) from the three parts of kenaf stems supported this scenario in different manners. The contents of these phytohormones were differentially elevated (ABA, Figure 7a; GA4, Figure 7e) or repressed (JA, SA, and IAA, Figure 7b–d, respectively) amongst the three stem parts, under salt stress compared with normal conditions. Collectively (Figure 7f), the overall contents variation of the three stress hormones (ABA, JA, and SA, Figure 7a–c, respectively) in kenaf stems was mainly contributed by that from the top and/or middle parts, whilst the contribution for GA4 was obtained from the middle and bottom parts (Figure 7e,f). Unlike the above-stated four phytohormones, the data suggested a minor NA-to-CO ratio for contents variation of IAA decrease (Figure 7d,f).

**Figure 8:**
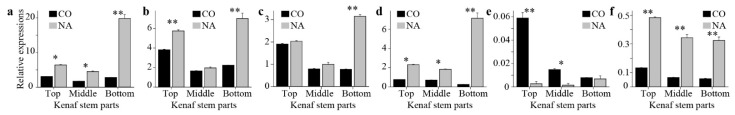
Relative expression levels of several unigenes. The relative expressions of *PAL* (**a**), *C4 H* (**b**), *CCR* (**c**), *F5 H* (**d**), *YUC* (**e**), and *AAO3* (**f**) genes from the three kenaf stems at the normal (CO) and stress (NA) conditions were respectively displayed. The *GAPDH* gene (Cluster-20656.100749) was selected as reference control for expression calculation. Primers and representative IDs for these six unigenes were listed in Table S5. *PAL*, phenylalanine ammonia lyase; *C4 H*, cinnamate 4 hydroxylase; *CCR*, cinnamoyl-CoA reductase; *F5 H*, ferulate 5 hydroxylase; *YUC*, flavin monooxygenases *YUCCA*; *AAO*, aldehyde oxidases. The * or ** indicate the significant difference at threshold of 0.05 or 0.01 under Student’s *t*-test, respectively.

To substantiate the connections between the above-stated transcriptome and the metabolome data, the relative expression levels for corresponding key genes involved in the lignin and designated phytohormone biosynthesis were quantified, which showed that the phenylpropanoid pathway genes were elevated under salinity stress, especially for the bottom parts of kenaf stems (Figure 8a–d). This is consistent with the transcriptomic output (Figure 3b) but contradictory against our intuition since the lignification was suppressed under salt stress (Figure 5). Meanwhile, the relative expression levels of the key genes responsible for auxin (Figure 8e) and ABA (Figure 8f) were universally consistent with the relative levels of respective metabolites (Figure 7a,d).

## 3. Discussion

Kenaf is an indispensable multi-purpose fiber crop [12], with only a few researches probing the molecular mechanisms when this species encounters adverse environmental conditions [13,16,23,24]. Especially, the molecular mechanisms of kenaf seedlings under salt stress have been respectively surveyed at the transcriptomic [16] or proteomic levels [13]. However, to the best of our knowledge, no assessment of how the kenaf stems respond to the salt stress has been performed, and neither was the metabolomics as a newly emerged methodology introduced to profile the metabolic changes of any kenaf organisms against the adverse conditions. In the current study, experimental data regarding the physiological traits (Figure 5), transcriptomic changes (Figure 2 and Figure 3), qRT-PCR validations (Figure 8), and the overall metabolic alterations (Figure 4) have indicated the different kenaf stem parts reacted dissimilarly to the salt stress.

Our data suggested several inconsistencies among the above results or with previous reports, which came primarily from the varied developmental status of corresponding kenaf stem parts. Indeed, as displayed by the stem slices (Figure 5), no lignification was observed except for the vessels in the top stem part. The xylems were similarly formed and seldom retarded by the stress in the middle, and the lignified proportions, as well as the fiber formations, have been significantly suppressed at the bottom. Overall, the stem diameter was shrunk (Figure 5), with numerous gene expressions altered (Figure 2), lignin contents lowered, and phytohormones changed (Figure 6). While the phytohormones play intricated and sophisticated roles during the salt stress [25], our results have added to the notion that this participation is complicated. For instance, the overall ABA contents increased rapidly to regulate the salt stress response [26]. Our results indicated the augments varied amongst the stem parts (Figure 6b and Figure 7a). IAA as the main auxin form was decreased and resulted in retarded plant growth under adverse conditions [27], as was observed in our data (Figure 6c and Figure 7d). The bioactive form of GAs as growth hormones were supposed to be similarly depressed similar to that of auxin [28], while the current output implied a controversial trend for GA4 in kenaf stems (Figure 7e). For these disparities between our measurements of phytohormones and general reports, possible explanations could be the species-specific responses as previously deduced [25]. Meanwhile, the kenaf stems as phenotyping samples may add perplexity to this issue since numerous phytohormones are mainly biosynthesized in the young leaves [29] and then transported to long-distance organisms, such as roots, through the stem [30]. Therefore, the phytohormone concentrations are not only a representation of the status of the stem itself, but they may also reflect the regulation of the whole seedling.

Secondly, manifold metabolites may belong to the same phytohormone and exist with similar or inverse functions. For instance, the IAA is the main auxin, and the tens of derivatives may be its inactive, storage, antagonist, or precursor forms [31]. A glimpse at these chemo-decorates indicated that although the IAA has decreased to similar concentrations among the three kenaf stem parts (Figure 7d), they may differ in how to achieve such a status (Figure 4e). Likewise, the isopentenyladenine and zeatin derivatives are two major groups of active cytokinin [32]. They may separately derivatize from the same precursor adenine and correspondingly respond to the salt stress in the top and middle parts of the kenaf stem (Figure 4e). Hence, a more comprehensive detection of the full spectrum of the phytohormones would provide a more inclusive view regarding how specific phytohormone species mediate the stress responses of kenaf stems.

One possible way to evaluate the phytohormone homeostasis is by monitoring the relative expression levels of functional genes involved in the metabolism of these metabolites [25,33,34], which was similarly indicated in our data (Figure 8e,f). Meanwhile, the universally up-regulated phenylpropanoid pathway genes (Figure 8a–d) were coincident with the transcriptomic indication (Figure 3b) but were inconsistent with the retarded lignification of kenaf stems (Figure 5). One possible explanation could be that the significantly elevated flavonoid pathway (Figure 3a) that is located downstream of the phenylpropanoid pathway has distracted the metabolite stream away from forming lignin. Moreover, some specific flavonoid metabolites have been shown to inhibit the polar translocation of auxin, thus affecting the plant architecture [35]. Hence, downstream studies could possibly probe the existence of previously reported endogenous inhibition of polar auxin transport by a given flavonoid metabolite [35], which may connect the overall enriched flavonoid pathway genes in kenaf stems (Figure 2) with distinctively enriched IAA decorates from different stem parts (Figure 4e).

## 4. Materials and Methods

### 4.1. Plant Materials

Kenaf cultivar H368 was obtained from Professor Defang Li (Institute of Bast Fiber Crops, Chinese Academy of Agricultural Sciences). The growth conditions were set as day/night cycle of 16 h/8 h, at 28 °C/25 °C, respectively, with a relative humidity close to 60% and a light intensity of 700 μmol m^−2^ s^−1^. A pot culture experiment was performed, and each pot (15 cm height, 18 cm diameter) was filled with a soil mixture of the same weight (red soil: humus: vermiculite, 2:1:1, *v*/*v*/*v*). Each pot was watered by 250 mL 1/4 Hoagland nutrient solution every other day. When the height of the plant reached 55 cm, the kenaf seedlings will enter the fast-growing stage. During this period of time, the plant height will increase from 2.1 cm to 5.0 cm per day, and the kenaf seedlings are highly sensitive to salt stress. Based on preliminary measurements on the POD and SOD (data not shown), 1 mol/L NaCl was presented in the 1/4 Hoagland solution for the salinity treatment. The kenaf seedlings for each replicate were collected at 72 h after the first NaCl watering, then frozen in liquid nitrogen and stored at −80 °C for the following experiments.

### 4.2. RNA Extraction, Library Preparation, and Sequencing

The stems from three kenaf seedlings for each replicate were collected at 72 h after adding the NaCl into the 1/4 Hoagland nutrient solution, frozen in liquid nitrogen, and stored at −80 °C for the following experiments. RNA extraction was conducted using the TRIzol reagent (Invitrogen, Waltham, CA, USA) following the manufacturer’s protocol. RNA quality was checked by the gel electrophoresis and Agilent 2100 Bioanalyzer (Agilent, Santa Clara, CA, USA) before further processing. The cDNA libraries for each biological replicate of control (denoted as CO1 and CO2) and NaCl treatment (NA1 and NA2) were respectively constructed and subjected to sequencing on the Illumina Hiseq2000 platform. Briefly, poly-A mRNA was isolated from total RNA with magnetic oligo (dT) beads and fragmented into small pieces. The double-stranded cDNA was synthesized using the SuperScript Double-Stranded cDNA Synthesis kit (Invitrogen, Waltham, CA, USA) with a random hexamer (N6) primer (Illumina, San Diego, CA, USA). After end-repair and phosphorylation using T4 DNA polymerase, Klenow DNA polymerase, and T4 polynucleotide kinase, these cDNA fragments were ligated with Illumina paired-end adapters to the ends of these fragments using T4 DNA ligase. The cDNA library was constructed with a fragment length of 200 bp ± 25 bp and then sequenced on a PE flow cell using an Illumina Hiseq2000 sequencing platform.

### 4.3. Data Assembly and Annotation

Raw data (raw reads) were first processed by removing the reads containing adapter sequences, poly-N, and low-quality reads from the raw data to generate clean data (clean reads). All downstream analyses were based on clean data of high quality. Transcripts were de novo assembled using Trinity [36] under default parameters and then further clustered into unigenes using the Corset [37] software. Gene function for unigenes was, respectively, annotated using information listed in Table S5.

### 4.4. Quantification of Gene Expression Levels and Differential Expression Analysis

The gene expression levels of each sample were estimated by mapping the clean reads onto the assembled transcriptome, using the RSEM (RNA-Seq by Expectation Maximization) software v1.2.15 (University of Wisconsin-Madison, Madison, WI, USA) [38] under default parameters. The mapped read counts were then transformed to FPKM (expected number of fragments per kilobase of transcript sequence per millions base pairs sequenced) values to evaluate the relative expression levels amongst different unigenes [39]. Differential expression analysis between the control and the salt stress conditions was performed using the DESeq R package version 1.12.0 [40], with threshold of padj < 0.05. GO enrichment analysis of the differentially expressed genes (DEGs) was implemented using GOseq R packages based on Wallenius non-central hypergeometric distribution [41], whilst the KEGG enrichment was conducted using the KOBAS software version 2.0.12 [42].

### 4.5. Quantitative Reverse Transcription PCR (qRT-PCR)

Total RNA was extracted from the whole seedlings subjected to normal (CO) or stress (NA) conditions using the RNAqueous Total RNA Isolation Kit (Ambion, Austin, TX, USA). Afterward, cDNA was reverse transcribed using the HiScrip II Q RT SuperMix for qPCR (Vazyme, Nanjing, China). The resulting cDNA was used as a template for RT-qPCR after being diluted 10-fold with sterile water. The GAPDH gene generated from current transcriptome sequencing (Cluster-20656.100749) was used as internal control gene. The qRT-PCR was performed in a LightCycler 480 II Real-Time PCR Detection System (Roche Ltd., Santa Clara, CA, USA). The 20 μL reaction mixture contained 10 μL ChamQ SYBR qPCR Master Mix (Vazyme, Nanjing, China), 2 μL cDNA template (approximately 100 ng of total RNA), and 0.5 μM of each forward and reverse primers (Table S6). The amplification parameters were as follows: 95 °C for 30 s followed by 40 cycles of 95 °C for 10 s and 60 °C for 30 s. Three independent experiments were carried out to ensure the reproductivity of qRT-PCR results. The relative expression levels were calculated using the 2^−ΔΔCT^ method [43].

### 4.6. Measurements of Lignin Monomers

The measuring method was previously established [44]. Briefly, 0.05 g of dried and finely ground samples were weighed and dissolved in the mixture 6 mL of 2 mol/L NaOH and 0.8 mL of nitrobenzene, heated to 100 °C overnight, and then cooled to room temperature. Samples were transferred to a 15 mL tube and extracted with ethyl acetate three times. The aqueous phase was adjusted to pH value at two, followed by an additional extraction with ethyl acetate three times. The organic phases were combined and dried with nitrogen gas and dissolved into methanol to a final volume of 0.5 mL. The liquid was filtered by 0.22 μm membrane and then subjected to measurement by HPLC at the wavelength of 290 nm.

### 4.7. Generation of Stem Slices

After subjected to the salinity stress, the 5 cm lengths of stems from the top, middle, and bottom were respectively cut and applied for stem slicing (seven seedlings for NA samples and three seedlings for CO samples), metabolic profiling (four seedlings each, same below) and quantitative real-time amplification (qRT-PCR). At least five slices were obtained from each part of stems and were respectively fixed in FAA solution (10% formaldehyde, 50% ethanol, and 5% acetic acid in deionized water) overnight at 4 °C, vacuumed, sectioned, and stained by the safranine-fast green method [45]. Briefly, the slices were rinsed in xylene, cleared in ethanol, and then hydrated in graded ethanol series before running tap water. Then the fast-green and the safranin staining solutions were respectively applied, with a fast wash of the slices in 1% acetic acid and then running water after each stain. Finally, the slices were dehydrated in ethanol and cleared in xylene.

### 4.8. Metabolic Profiling of Kenaf Stems

The above-mentioned kenaf stem parts were collected, snap-frozen in liquid nitrogen, freeze dried, and then pulverized into fine powder. In total, 50 mg of powders were weighed for each sample, and 500 μL of 70% methanol (HPLC grade) were added for metabolite extraction, respectively. The extraction processes were previously described [20], and the filtered liquids were subjected to the measurements. A widely targeted metabolic profiling method [19] was utilized, using the previously established metabolite library and machine parameters [20].

### 4.9. Statistical Analyses

Statistical analyses were performed with SPSS software version 22.0 (SPSS, Chicago, IL, USA). Error bars represent standard deviation (S.D.). Student’s *t*-test was applied to analyze whether the significance existed between the stress (NA) and normal (CO) conditions, and one to three asterisks denote significant differences between corresponding controls and treatments at thresholds of 0.05, 0.01, and 0.001, respectively. Metabolic data presentations were achieved using the online tool [46].

## 5. Conclusions

We have presented a systematic evaluation of how kenaf reacted to the salt stress at the physiological, transcriptomic, and metabolic levels, which suggested this adverse condition could retard kenaf growth, shrink fiber formation, and lignification. During this process, the growth phytohormones such as IAA were repressed, whereas the stress hormones, including ABA, were elevated. However, the transcriptome data and the qRT-PCR output did not always perfectly agree with the metabolite alterations, suggesting the complexity of secondary metabolic pathways. One possible explanation is the counterbalance among several deviations of metabolic routes for a certain metabolite, and it is hard to simultaneously evaluate the enormous nodes for these metabolic pathways. Therefore, although plentiful data have been carried out in the current study, more evidences are still needed to further probe how the metabolites reacted to the salt stress and how the metabolic adjustment finally resulted in the physiological traits.

## Data Availability

The transcriptomic data have been submitted to the GenBank database (SRR9613936 to SRR9613939).

## Supplementary Materials

The following are available online at https://www.mdpi.com/article/10.3390/plants11111448/s1, Figure S1: Correlation of the sample repeats subjected to the transcriptomic sequencing, Figure S2: Functional annotation of transcriptomic unigenes. The annotation was performed against the GO (a), KOG (b), and KEGG (c) databases, Table S1: The overall information for current transcriptome sequencing results, Table S2: Annotation of unigenes against seven databases, Table S3: The overall glimpse of metabolites detected in the current study, Table S4: Differentially enriched metabolites in each of the three kenaf stem parts, Table S5: Brief information of how the unigenes were annotated against the seven databases, Table S6: Primers for the randomly selected unigenes that conducting qRT-PCR.
