# Supplementary figures and images for: Transcriptomic and Metabolic Profiling of Kenaf Stems under Salinity Stress
Source: Plants (Basel). 2022 May 29;11(11):1448. doi: 10.3390/plants11111448 (PMC9182824; doi:10.3390/plants11111448)

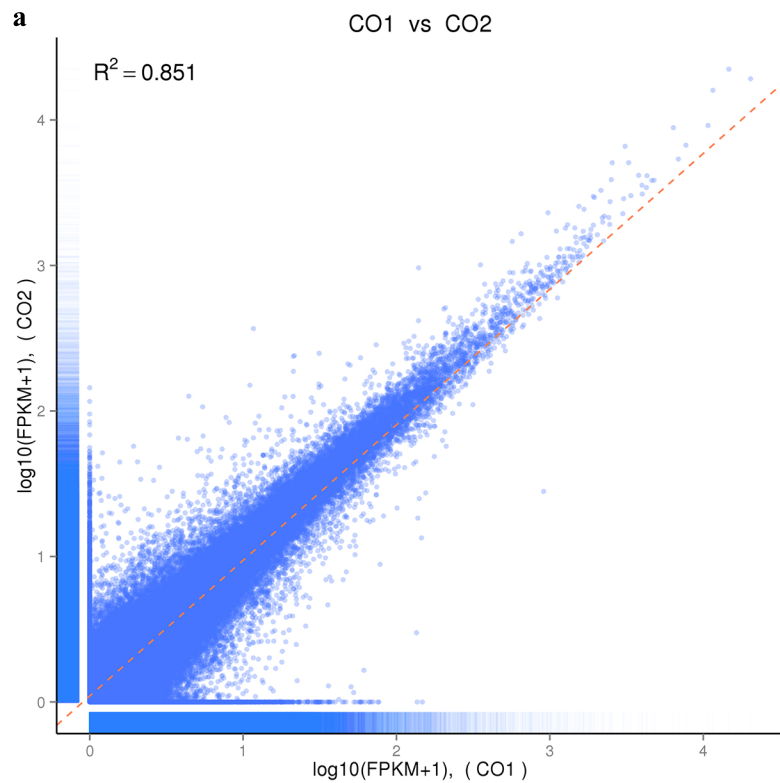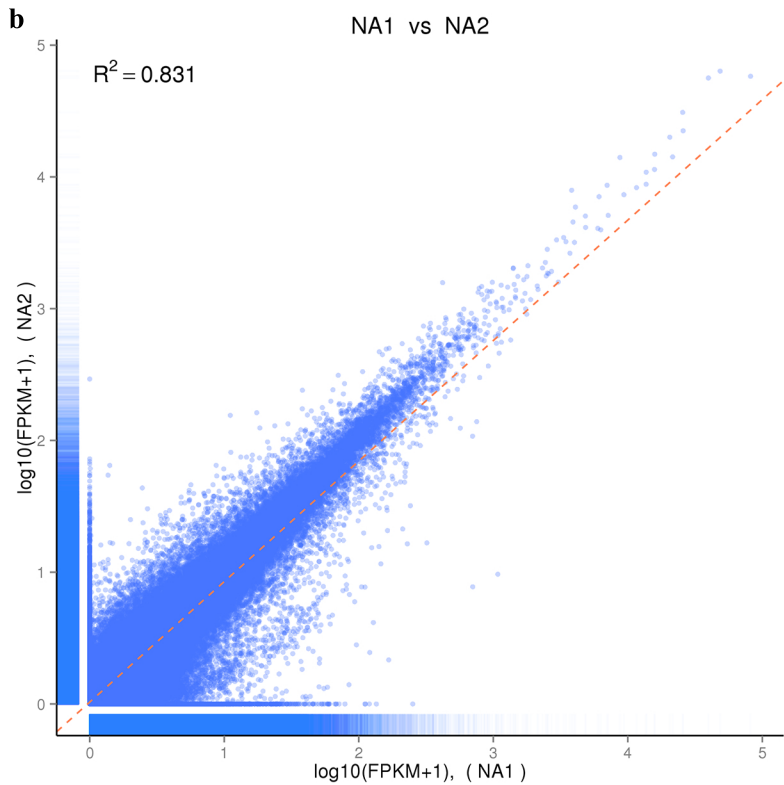

Supplement: Supplementary file 1 [file plants-11-01448-s001.zip › Figure S1.pdf]

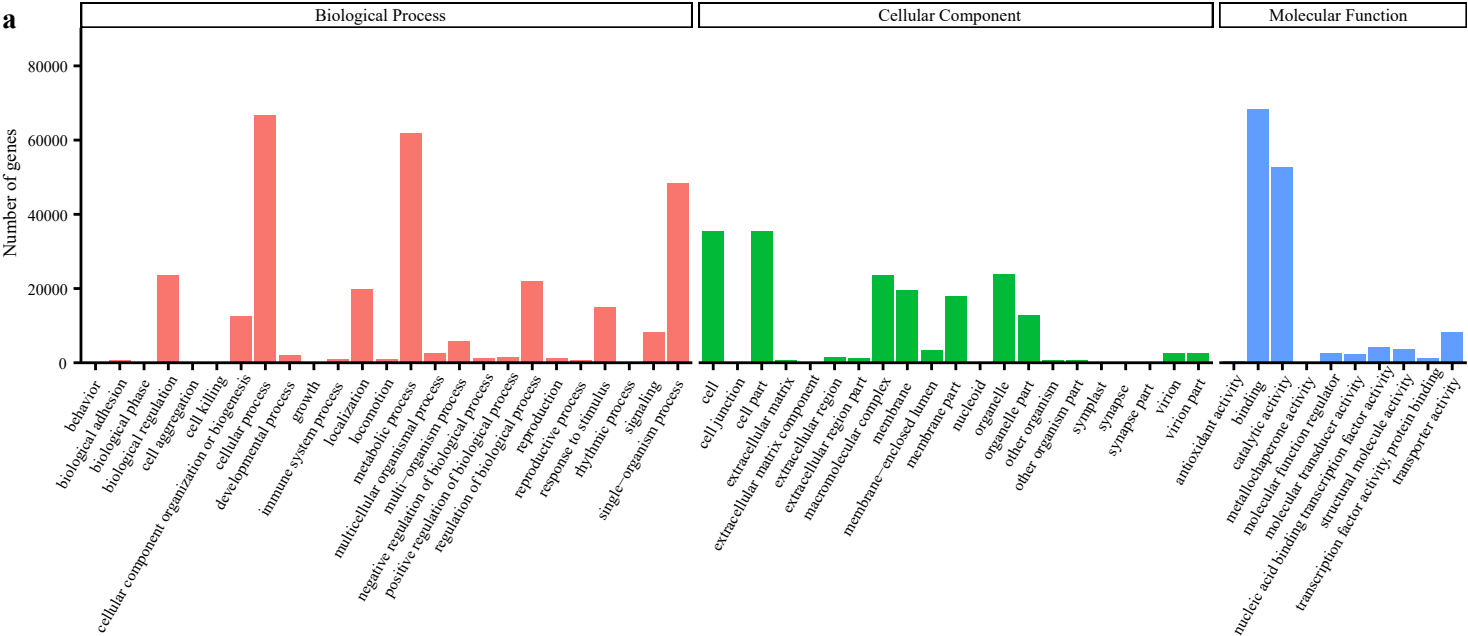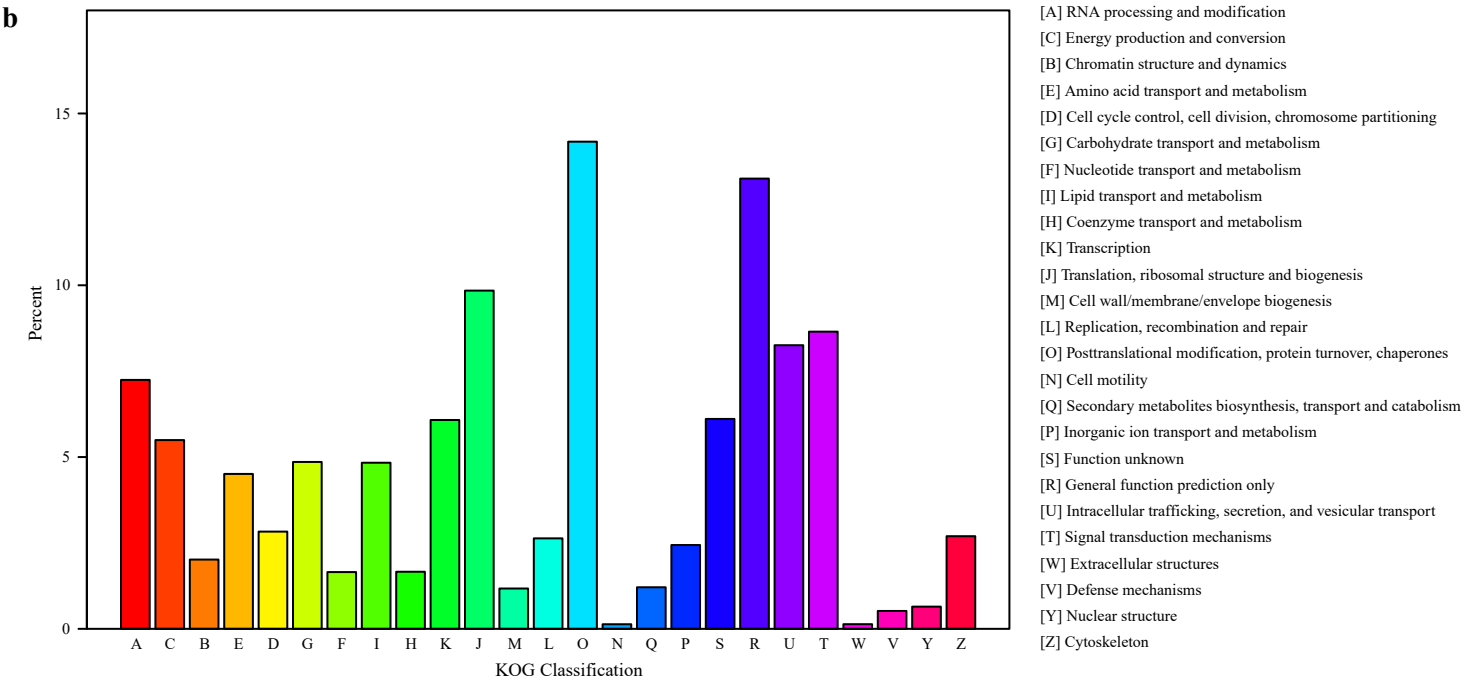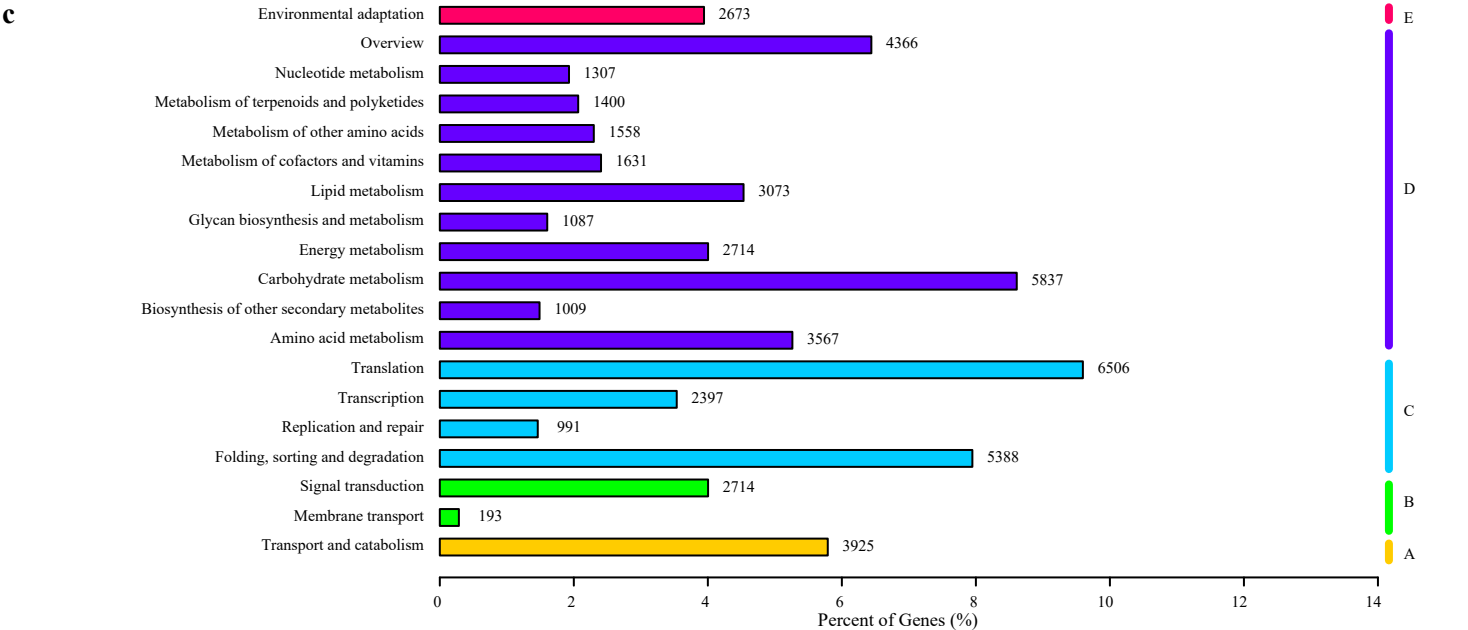

Supplement: Supplementary file 1 [file plants-11-01448-s001.zip › Figure S2.pdf]
